# Sustainable Greenhouse Tomato Production: Benefits of Inoculation With Arbuscular Mycorrhizal Fungi Under Low Nitrogen and Phosphorus Conditions

**DOI:** 10.1002/pei3.70058

**Published:** 2025-11-23

**Authors:** Fazal Ullah, Faisal Zaman, Muhammad Ishfaq, Habib Ullah, Chunwei Wang, Li Zhifang, Christoph‐Martin Geilfus

**Affiliations:** ^1^ Beijing Key Laboratory of Growth and Developmental Regulation for Protected Vegetable Crops College of Horticulture, China Agricultural University Beijing China; ^2^ State Key Laboratory of Nutrient Use and Management, College of Resources and Environmental Sciences, National Academy of Agriculture Green Development, Key Laboratory of Plant‐Soil Interactions China Agricultural University Beijing China; ^3^ College of Life Sciences and Oceanography Shenzhen University Shenzhen China; ^4^ College of Chemical Engineering Tianjin University Tianjin China; ^5^ Department of Chemistry Govt Post Graduated College Mardan, Abdul Wali Khan University Mardan KPK Pakistan; ^6^ Beijing Cuihu Agricultural Technology Co. Ltd Beijing China; ^7^ Department of Soil Science and Plant Nutrition Hochschule Geisenheim University Geisenheim Germany

**Keywords:** AMF inoculation, chlorophyll, fruit yield, mineral nutrients, mycorrhizal colonization, plant‐fungi interactions, symbiosis

## Abstract

The effects of overused chemical fertilizers, which threaten soil, plant, and human health, have always remained a topic of interest in theory and practice, emphasizing the judicious use of mineral nutrients. This study was aimed at reducing the harmful effects of excessive chemical fertilizer application and at exploring alternative approaches that can improve soil fertility without environmental and health damage. The experimental design involved a controlled greenhouse setup where tomato cultivars were inoculated with different AMF species under varying nitrogen (N) and phosphorus (P) doses. The tomato cultivars Rio Grande and Nadir were inoculated with arbuscular mycorrhizal fungi species, including *Glomus claroideum*, *Glomus etunicatum*, *Glomus fasciculatum*, and *Glomus mosseae*—within a commercial greenhouse. This study aimed to evaluate the potential effects of these fungi on tomato growth physiology, yield, and fruit quality when subjected to varying doses of N and *P. Glomus mosseae* significantly increased plant height by 14%, stem diameter by 22.25%, dry matter by 23.59%, yield by 38.57%, N uptake by 16.40%, P uptake by 37.5%, potassium (K) uptake by 18.55%, chlorophyll a (Chl a) content by 15.18%, and chlorophyll b (Chl b) content by 25.19% when compared to untreated controls. Additionally, *Glomus mosseae* improved fruit diameter by 9.98%, fruit firmness by 18.45%, juice content by 15.20%, titratable acidity (TA) by 10.42%, and ascorbic acid concentration by 16.75%. The interaction between the N and P levels of 140:42 mg L^−1^ and the arbuscular mycorrhizal fungus (AMF) species *Glomus mosseae* resulted in the highest improvement in growth, yield, and fruit quality‐related traits. Among the cultivars, Rio Grande exhibited the greatest root colonization, plant dry matter content, N, P, K uptake, plant height, Chl a, Chl b, and yield when compared to the control. In contrast, cultivar Nadir showed the highest stem diameter, fruit size, firmness, ascorbic acid, fruit juice contents, and TA. This study recommends that AMF inoculation in combination with a low N and P supply can be promising for improving tomato growth, productivity, and fruit quality on a commercial scale with minimum threats to the environment and human health. This study suggests the exploration of long‐term sustainability and scalability of AMF inoculation methods in diverse agricultural settings.

**Abbreviations**
C: Control
Chl a: Chlorophyll a
Chl b: Chlorophyll b
CV: Cultivar
FW: Fresh weight
GC: *Glomus claroideum*
GE: *Glomus etunicatum*
GF: *Glomus fasciculatum*
GM: *Glomus mosseae*
K: Potassium
N: Nitrogen
P: Phosphorus
Sig: Significance

## 1 Introduction

Arbuscular mycorrhizal fungi (AMF) benefit both themselves and the plants by facilitating the exchange of mineral nutrients for carbon; the fungi provide essential mineral nutrients to the plant in return for photosynthetic carbon. Mycorrhizal association develops when fungi and plant roots form a symbiotic relationship (Hodge and Storer 2015). AMF are the type of plant symbiosis that occurs the most frequently and extensively. The AMF improve the plant's efficiency in taking up nitrogen (N) and phosphorus (P) (Smith and Smith 2011), and it can also enhance the plant's capacity to absorb other mineral nutrients (Casieri et al. 2012; Cheaib et al. 2025).

AMF symbiosis is a more organized activity that occurs only when nutrient transfer benefits both partners via a mutually beneficial reward system. This particular form of symbiosis can only take place under specific conditions. For example, arbuscules accumulate polyphosphate and quickly degenerate in *Medicago truncatula* mutants carrying a mutation in the AMF‐specific phosphate transporter 4 (MtPT4) gene (Che et al. 2022), indicating that phosphate transport to cortical cells regulates the maintenance of symbiosis. On the other hand, the provision of carbon (C) to the fungus boosts N and P uptake and transport, and this stimulation is related to alterations in fungal gene expression (Fellbaum et al. 2012; Ahmed et al. 2025). However, N and P are essential for the nutrient transfer process during AMF symbiosis. Mycelium transports phosphate as polyphosphate and then liberates it in the arbuscules through polyphosphates (Funamoto et al. 2007; Behera et al. 2024). Nitrogen is transported in the extraradical hyphae as arginine (Tian et al. 2010; Qiu et al. 2025), which binds to polyphosphate and is thus involved in Pi transport (Pellegrino et al. 2011; Zhang et al. 2025). The breakdown of arginine in the intraradical mycelium releases ammonium, which at that time may transfer to the host plant (Tian et al. 2010; Qiu et al. 2025). The organized transport of P and N in AMF symbiosis improves nutrient exchange, allowing plants to flourish in nutrient‐deficient soils by improving nutrient uptake efficiency (Funamoto et al. 2007; Mehmood et al. 2025). This symbiosis reduces the dependency on chemical fertilizers and increases overall plant productivity (Tian et al. 2010; Qiu et al. 2025). According to Pellegrino et al. (2011), how AMF inoculums are applied to soil and the crop type impact its effectiveness in crop production. In general, plant roots that are inoculated with AMF can be more effective in obtaining nutrients and water from the soil, which results in better plant performance (Delavaux et al. 2017; Ullah, Ullah, Ishfaq, Gul, et al. 2023; Ullah, Ullah, Ishfaq, Khan, et al. 2023). AMF positively affects the performance of both fruits and bulbous plants and plant water use efficiency. This effect is also observed in vegetable crops, such as watermelon, and onion (Tang et al. 2022). For example, plants treated with AMF show higher growth, increased fruit and bulb yield, and improved water use efficiency under water‐stressed conditions (Santander et al. 2017; Delavaux et al. 2017; Kanwal et al. 2024). A study in *Medicago truncatula* clarified the role of nutrient supplementation and P‐N crosstalk in promoting a positive interactive support response in cortical cells (Smith and Smith 2011; Shen et al. 2025). Plant P influences AMF symbiosis (Smith and Smith 2011), and systemic signaling controls root colonization in low‐P environments, as demonstrated in split‐root tests (Che et al. 2022; Alhammad et al. 2023). In the mtpt4 mutant defective in AMF symbiosis, N deficiency reduces premature arbuscular decay and root colonization initiation (Balzergue et al. 2011; Ali et al. 2023). Plants with both N and P deficiencies exhibit closely related symptoms, such as stunted growth, increased starch and anthocyanin content, and more (Ishfaq, Tam, et al. 2025). Low plant N can sometimes reduce mycorrhizal development, but not always (Bunn et al. 2024). Tomato plants require sufficient amounts of N and P to grow and produce high‐quality fruits, and nutrient deficiencies can limit their productivity (Ishfaq et al. 2021; Ullah, Ullah, Ishfaq, Gul, et al. 2023; Ishfaq, Wang, et al. 2023, 2025).

The excessive chemical fertilizer inputs (i.e., N and P) can negatively affect soil health and environmental quality (Wakeel and Ishfaq 2022; Ishfaq, Kiran, et al. 2022; Yokamo et al. 2023; Wang et al. 2025); therefore, exploring alternative strategies to increase nutrient uptake and photosynthetic activity in tomato plants is essential for sustainable agriculture. There is limited understanding of the long‐term impact of AMF on crop productivity under various nutrient stress conditions, particularly mild N and P deficiencies. Most studies focus on severe deficiencies, leaving a gap in understanding the effects of nutrient limitations. In this study, we aimed to explore the potential of AMF to handle the mild deficiency stress of N and P in tomato plants. We hypothesized that the application of AMF can be promising in plant growth, nutrient uptake, and photosynthetic activity under mild deficiency stress of N and P. The present study's findings offer an eco‐friendly solution, reduce over‐reliance on chemical fertilizers, and improve the growth and productivity of tomatoes.

## 2 Materials and Methods

### 2.1 Plant Material and AMF Species

The experiment was conducted during 2021–2022 in a controlled greenhouse (25°C ± 2°C temperature, relative humidity 60%, 14 h' light/10 h dark) at Kalar Kahar (32.73 N, 72.82 E), in the Punjab of Pakistan. The research design was a randomized complete block (RCBD) with three factors and four replications. Ten plants per treatment were grown. Factors were AMF species, N:P levels, and tomato cultivars. Commercial AMF species (*Glomus claroideum* (GC) strain *BEG23*, *Glomus etunicatum* (GE) strain *CHI‐10*, *Glomus fasciculatum* (GF) strain *IES‐1*, and *Glomus mosseae* (GM) strain *BEG 54*) were bought from an Indonesian company “Agrow isata” (99 spore/100 g, isolated strains of Indonesian soils). These four AMF species are beneficial under different environmental conditions (such as drought, salinity, and nutrient‐poor soils). These species are compatible with a variety of plant hosts, making them versatile for different crops or ecological systems, and help optimize plant growth, nutrient uptake, and soil health. Two tomato cultivars, namely, Rio Grande and Nadir, were used that were provided by the National Agricultural Research Council (NARC) Islamabad, Pakistan. Their nursery was raised in peat and vermiculite substrate (2:1) before transplantation. AMF species were applied to the plant roots first at the time of seed sowing and second just after transplantation. The N and P application rates were 200:60, 140:42, and 100:30 mg L^−1^. The nutrient solution was prepared using different fertilizer sources dissolved in distilled water before application, and details are mentioned in Table 1.

**TABLE 1:** Composition of nutrient solution applied to tomato plants.

	Fertilizer type	Concentration (g/100 L)
A	CO(NH_2_)_2_	434.8 g
	CO(NH_2_)_2_	304.3 g
	CO(NH_2_)_2_	217.4 g
B	MgSO_4_. 7H_2_O	230 g
	TSP	300 g
	TSP	210 g
	TSP	150 g
C	H_3_BO_3_	1.42 g
	MnSO_4_. H_2_O	1.69 g
	ZnSO_4_. 7H_2_O	1.30 g
	CuSO_4_. 5H_2_O	0.19 g
	EDTA‐FeNa. 3H_2_O	6.80 g

Abbreviations: EDTA, Ethylenediaminetetraacetic acid; TSP, triple superphosphate.

### 2.2 Preparation Before Sowing

The peat soil and vermiculite (1–3 mm) were mixed evenly 2:1 (peat soil was crushed). The prepared substrate was placed in plastic bags. The inoculation of AMF was carried out by adding 25 g of commercial AMF inoculum per kg of growing media of each species and was mixed with the peat and vermiculite mixture thoroughly, ensuring even distribution before planting (Ramírez Caro et al. 2011). The tomato seeds were sown in the treated growing medium according to the experimental design.

### 2.3 Sowing and Daily Management

Tomato seedlings were grown for 2 months in the nursery trays before transplantation. Each seedling tray contained 20 holes (10 for Nadir and 10 for Rio Grande cultivar). Seeds were sown and covered lightly until germination. Every other day, 100 mL of nutrient solution was added into each hole. The basic dose of nutrient solutions given to the seedlings is listed in Table 1. The position of the trays was changed every day to ensure the even distribution of light.

In the first 20 days, each tray was irrigated with 1100 mL each time. In the middle of 20 days, each plug was irrigated with 1200 mL each time. In the last 20 days, each tray was irrigated with 1300 mL each time. Before transplanting, 25 g of commercial AMF inoculum per kg of growing media of each species was applied. After 2 months of sowing, the seedlings were transplanted into their regular seed beds. The roots were covered with growth medium (peat + vermiculite + soil with 1:1:2). After transplantation, the same nutrient solution was applied every 1–2 weeks (Table 1).

### 2.4 Plant Growth Measurements

Plant growth and physiological parameters were recorded just before harvesting.

#### 2.4.1 Determination of Height, Stem Diameter, and Dry Matter Content

Six plants per treatment group were randomly picked and measured in centimeters using a measuring tape, and the average was calculated. Stem diameters of five random plants per experimental unit were measured with a vernier caliper (Starrett 125 Series). For dry biomass, the total oven‐dried weight of the leaves, stems, and roots from each sampling area was recorded and computed as follows (Franco and Rinne 2023):

$$\mathrm{Dry}\;\text{matter}\;\left(\%\right)=\frac{\mathrm{Dry}\;\text{weight}}{\text{Fresh weight}}\times 100$$

#### 2.4.2 Measurement of Root Colonization

**FIGURE 1:**
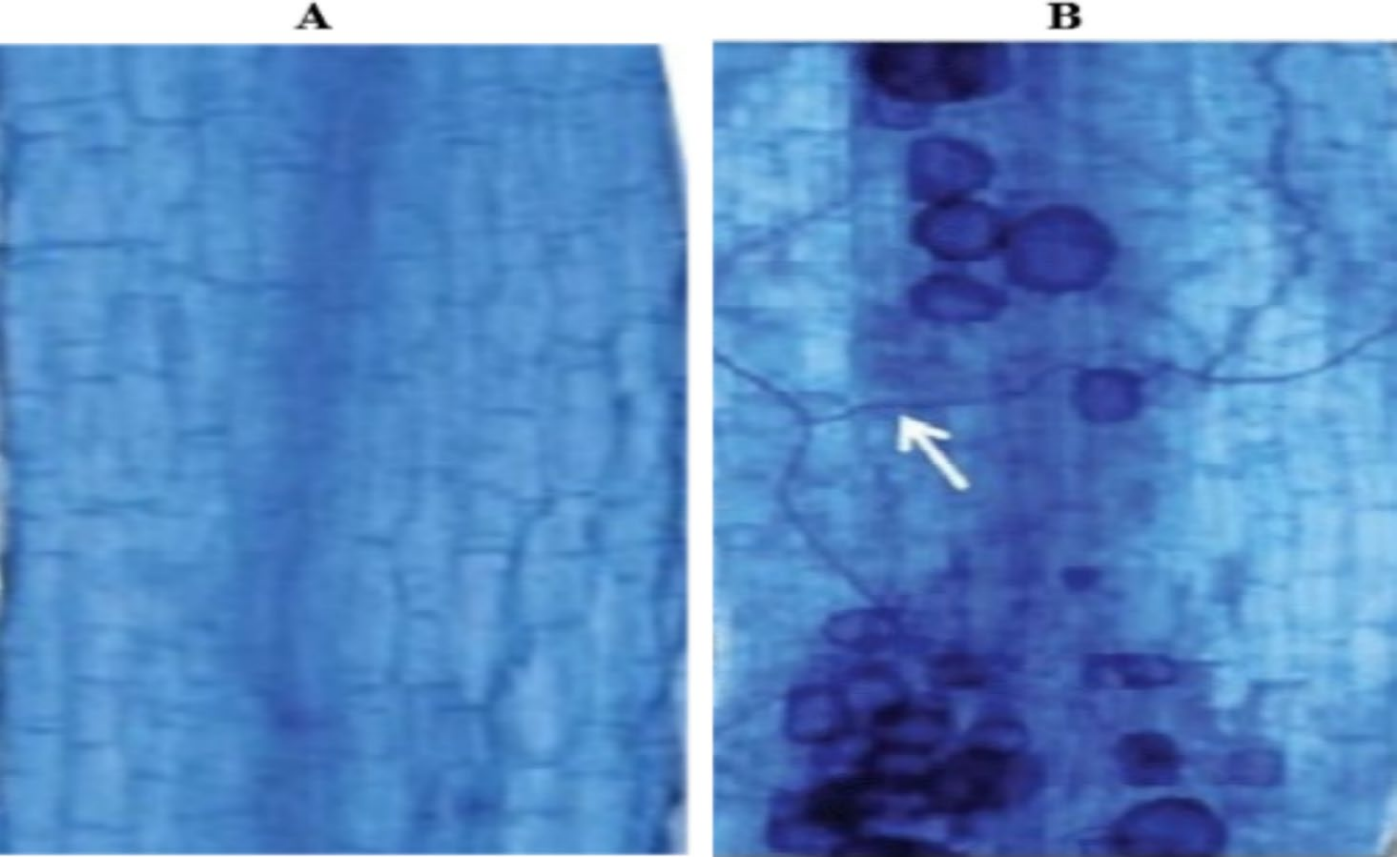
Observation of root colonization under the compound microscope at ×200 magnification (A) non‐inoculated tomato root (B) inoculated tomato root. AMF, arbuscular mycorrhizal fungi.

The root samples were rinsed gently with tap water to remove any attached soil particles. Afterward, they were cut into pieces of 1 cm in size. The root segments were first cleaned by immersing them in a 10% KOH (w/v) solution and heating them in a water bath for 30 min at 90°C (Giovannetti and Mosse 1980). Then, the roots were washed several times with DW. Next, a freshly prepared alkaline H_2_O_2_ solution was used for 60 min to bleach the roots. This solution was prepared by mixing 3 mL of NH_4_OH_2_ with 30 mL of 10% H_2_O_2_ and 567 mL of DW. After bleaching, the roots were rinsed with DW. To make the root samples acidic, they were immersed in a 2% HCl (v/v) solution for 5 min. The roots were then rinsed twice with DW. The cleaned roots were stained with trypan blue in lactoglycerol (0.05%) and incubated for 45 min at 90°C. After washing the root samples with water, they were placed on glass slides and observed under a compound microscope (Figure 1). The amount of colonization on each root was determined, as reported earlier (Giovannetti and Mosse 1980). Briefly, this method involves gently squeezing the stained root segment onto a microscope slide and covering it with a coverslip. From each plant, fifty pieces were analyzed with the utmost care and precision, ensuring the technique's reliable application. The following formula was used to calculate the percentage (%) of mycorrhizal colonization:

$$\text{Root colonization}\;\left(\%\right)=\frac{\mathrm{No}.\;\text{of colonized roots}}{\text{Total}\;\mathrm{No}.\;\text{of inspected roots}}\times 100$$

#### 2.4.3 Mineral Elements Analysis

After harvesting, the mineral elements were determined through the ashing method. The ashing process was briefly performed in an oven by heating to 500°C for 6 h. The spectrophotometer and flame photometer (Systronic 129c) were used to analyze P and K (AOAC 1980), and the Kjeldahl apparatus was used to analyze N in the plant tissues (Yoshida et al. 1976). For mineral nutrients, the data were presented on a dry weight basis.

#### 2.4.4 Determination of Chlorophyll Contents

Sample of tomato leaves (0.2 g) was extracted thrice by using 80% acetone. After filtering the mixture, the supernatants were mixed. The total volume was then increased to 25 mL. The absorbance was recorded at 470, 663, 652, and 645 nm using a UV–VIS spectrophotometer (Genesis 20). Chlorophyll (Chl) a and b were determined in mg g^−1^ of the extract by following the formula (Cornea‐Cipcigan et al. 2022; Yang et al. 2025).

$$\mathrm{Chl}\;a=\left(11.75\times A_{663}–2.35\times A_{645}\right)\times V/g$$

$$\mathrm{Chl}\;b=\left(18.61\times A_{645}–3.96\times A_{663}\right)\times V/g$$

$$\text{Total}\;\mathrm{Chl}=\left(27.8\times A_{652}\right)$$

Whereas *V* is the volume of extract (mL), g is the weight of the sample, and *A*_645_ and *A*_663_ are optical densities at a particular wavelength.

#### 2.4.5 Tomato Yield

After the fruits reached physiological maturity, tomato fruit samples were collected by harvesting six plants per plot three times weekly (Ishfaq, Wang, et al. 2025). The fruit weight of each plant was measured using a high‐accuracy weighing scale.

#### 2.4.6 Measurement of Fruit Diameter (mm)

A Vernier caliper (Starrett 125 Series Vernier Caliper) was used to identify the fruit diameter of five plants per experimental plot in millimeters (mm), and then the mean was measured as described earlier (Ishfaq, Wang, et al. 2025).

#### 2.4.7 Measurement of Fruit Firmness (kg cm^−2^)

The fruit firmness of tomato fruit was assessed using a penetrometer (specifically, model FT 011 with an 8 mm probe). Five readings were taken per treatment in each replication, and the means were calculated from these readings (Plocharski et al. 2000).

#### 2.4.8 Measurement of Fruit Juice Content (%)

To calculate the juice percentage in each fruit sample, we measured the sample's weight before and after extracting the juice. The difference between these two weights was used to determine the weight of the extracted juice. The following formula was used to calculate the percentage of juice (Arendse et al. 2021):

$$\text{Percent juice content}=\frac{\text{Average juice weight}}{\text{Average fruit weight}}\times 100$$

#### 2.4.9 Measurement of Titratable Acidity (TA %)

The acidity level was measured using the neutralization reaction procedure according to the guidelines of A.O.A.C (1990). The standard 0.1 N sodium hydroxide (NaOH) solution was used to titrate the sample of unknown acidity, and the endpoint was indicated using phenolphthalein. The process involved taking 10 mL of the unknown solution, diluting it with water to make a 100 mL volume, and then titrating 10 mL of the diluted sample with the phenolphthalein indicator. When the pink color appeared for 15 s, the reading was recorded. Three measurements were noted for each treatment, and the acidity was calculated as follows:

$$\text{Percent titratable acidity}=\frac{T\times F\times N\times 100}{D\times S}\times 100$$

F is the constant acid factor for the primary acid in the tomato fruit, which is citric acid, is 0.0067; D is the sample used for dilution (mL); *N* is the Normality of NaOH; S is the diluted sample used for titration (mL), and T is the NaOH taken from the burette (mL).

#### 2.4.10 Measurement of Ascorbic Acid (mg 100 g^−1^)

The ascorbic acid content of all treatments was analyzed as reported by A.O.A.C. (1990). In brief, the dye solution was prepared by dissolving 50 mg of 2, 6‐dichloroindophenol dye and 42 mg of sodium bicarbonate (NaHCO_3_) in a 200 mL beaker filled with hot DW. After 30 min of stirring, the mixture was added to a volumetric flask until it reached 250 mL. To standardize the dye solution, 50 mg of ascorbic acid was thoroughly dissolved in 50 mL of oxalic acid solution (0.4%). Followed by a conical flask containing 2 mL of this solution, which was then titrated with the dye solution until a distinct pink color was observed. A formula was used to calculate the coloring factor, which was added to the ascorbic acid content formula.

$$\mathrm{Dye}\;\text{factor}\left(F\right)=\frac{\mathrm{ml}\;\text{ascorbic acid slution}}{\mathrm{ml}\;\mathrm{dye}\;\text{solution used}}\times 100$$

Ten milliliters of tomato fruit juice were obtained and diluted to a final volume of one hundred milliliters using a 0.4% oxalic acid solution to identify the ascorbic acid concentration. Afterward, 10 mL of the diluted sample was placed in a conical flask and titrated using a dye solution until a pale pink color appeared. The level of ascorbic acid was calculated using a formula that considers the dye factor, the volume of dye solution used, the amount of tomato juice used for dilution, and the volume of the diluted sample used for titration.

$$\text{Ascorbic acid content}=\frac{F\times T\times 100}{D\times S}\times 100$$

F is the Dye factor; T is the Dye solution used in titration from the burette (mL); D is the Diluted sample used for titration (mL), and S is the Tomato juice used for dilution (mL).

### 2.5 Statistical Methods

The SPSS 25 software was used to carry out the statistical analysis. Data was analyzed using variance analysis (ANOVA). After three‐way ANOVA, the differences between the means across treatments were analyzed by the Tukey test at *p* ≤ 0.05. The results are presented as mean and maximum/minimum values.

## 3 Results

### 3.1 Effect on Growth, Physiology, Photosynthetic Pigments, and Yield of Tomato

Plant height, dry matter, stem diameter, N, P, K concentrations, Chl contents, AMF colonization, and the Rio Grande and Nadir yield were significantly (*p* ≤ 0.05) by AMF species. Among AMF species, *Glomus mosseae* showed the highest plant height, dry matter content, stem diameter, NPK, Chl contents, AMF colonization, and yield, followed by *Glomus fasciculatum* (Table 2). However, these morphological and physiological characteristics were lowest in the control treatment (Table 2).

**TABLE 2:** Effect of AMF species on tomato growth, mineral nutrient, Chl contents, and yield.

AMF species	Plant height (cm)	Stem diameter (mm)	Dry Matter (%)	Root Colonization (%)	N (%)	P (%)	K (%)	Chl a (mg g^−1^ FW)	Chl b (mg g^−1^ FW)	Yield (kg plant^−1^)
Control	55.76 ± 2.48 e	15.26 ± 1.39 e	10.37 ± 1.34 e	6.83 ± 1.38 e	2.84 ± 0.12 e	0.42 ± 0.04 e	2.79 ± 0.28 e	2.73 ± 0.29 e	1.47 ± 0.28 e	1.41 ± 0.21 e
*G Claroideum*	61.87 ± 3.17 d	17.77 ± 1.65 d	11.80 ± 1.10 d	64.23 ± 5.54 d	3.21 ± 0.22 d	0.53 ± 0.05 d	3.35 ± 0.29 d	3.16 ± 0.31 d	1.85 ± 0.19 d	1.83 ± 0.22 d
*G Etunicatum*	63.44 ± 3.40 c	18.79 ± 1.81 c	12.53 ± 0.85 c	68.21 ± 5.51 c	3.33 ± 0.23 c	0.63 ± 0.06 c	3.53 ± 0.28 c	3.26 ± 0.30 c	2.01 ± 0.17 c	2.00 ± 0.20 c
*G Fasciculatum*	65.60 ± 3.64 b	19.81 ± 1.73 b	13.31 ± 0.67 b	72.46 ± 5.38 b	3.45 ± 0.23 b	0.72 ± 0.04 b	3.72 ± 0.27 b	3.37 ± 0.32 b	2.16 ± 0.15 b	2.14 ± 0.19 b
*G Mosseae*	67.20 ± 3.92 a	20.85 ± 1.83 a	14.07 ± 0.68 a	76.59 ± 5.38 a	3.58 ± 0.24 a	0.82 ± 0.07 a	3.93 ± 0.29 a	3.49 ± 0.35 a	2.33 ± 0.17 a	2.30 ± 0.19 a

*Note:* The percentage of inoculated roots was used to calculate root colonization. The dry matter content was determined as a percentage of the shoot, leaves, and root's dry weight. N, P, and K concentrations were calculated as a percentage of their respective dry weights. The Chl contents were measured in terms of fresh weight. The data are presented as the means ± SD. The different letters show significant differences (*p* ≤ 0.05) among the treatments (*n* = 5).

Abbreviations: AMF, Arbuscular mycorrhizal fungi; Chl, chlorophyll; Control, without AMF‐inoculation; K, potassium; N, nitrogen; P, phosphorus.

In the case of N:P, the highest plant height, dry matter content, stem diameter, N, P, K, Chl contents, and yield were recorded in plants treated with 140:42 mg L^−1^ of N:P, whereas the lowest was recorded in plants treated with 100:30 mg L^−1^. The maximum AMF colonization was found in plants with an NP level of 100:30 mg L^−1^, whereas the lowest colonization was noted in plants with 200:60 mg L^−1^ (Table 3).

**TABLE 3:** Effect of NP levels on tomato growth, mineral nutrient, Chl contents, and yield.

N:P levels	Plant height (cm)	Stem diameter (mm)	Dry Matter (%)	Root Colonization (%)	N (%)	P (%)	K (%)	Chl a (mg g^−1^ FW)	Chl b (mg g^−1^ FW)	Yield (kg plant^−1^)
200:60 mg/L	62.10 ± 4.10 b	18.19 ± 1.41 b	11.90 ± 1.32 b	54.13 ± 5.40 c	3.30 ± 0.15 c	0.69 ± 0.09 b	3.47 ± 0.31 c	3.20 ± 0.34 b	2.00 ± 0.30 b	1.90 ± 0.31 b
140:42 mg/L	64.29 ± 3.21 a	19.29 ± 1.61 a	12.89 ± 1.68 a	57.55 ± 6.10 b	3.41 ± 0.20 a	0.76 ± 0.06 a	3.65 ± 0.30 b	3.33 ± 0.35 a	2.14 ± 0.25 a	2.10 ± 0.39 a
100:30 mg/L	60.93 ± 2.51 c	17.11 ± 1.54 c	11.05 ± 1.57 c	61.31 ± 6.30 a	3.22 ± 0.17 c	0.64 ± 0.08 c	3.28 ± 0.26 a	3.08 ± 0.33 c	1.83 ± 0.21 c	1.71 ± 0.30 c

*Note:* Dry matter content was determined by calculating the percentage of shoot, root, and leaf dry weights. Root colonization was quantified as the percentage of roots colonized. N, P, and K concentrations were calculated as dry weight percentages for each element. Chl contents were measured using fresh weight. The values are presented as means ± SD. The letters represent the significant treatment differences (*p* ≤ 0.05, *n* = 5).

Abbreviations: Chl, chlorophyll; K, potassium; N, nitrogen; P, phosphorus.

In the case of cultivars, Rio Grande showed higher plant height, N, K, Chl contents, AMF colonization, and yield than cultivar Nadir; however, Nadir showed a larger stem diameter than Rio Grande (Table 4).

**TABLE 4:** Effect of tomato cultivars on tomato growth, mineral nutrients, Chl contents, and yield.

Cultivars	Plant height (cm)	Stem diameter (mm)	Dry matter (%)	Root colonization (%)	N (%)	P (%)	K (%)	Chl a (mg g^−1^ FW)	Chl b (mg g^−1^ FW)	Yield (kg plant^−1^)
Rio grande	63.67 ± 3.12 a	17.62 ± 1.44 b	12.62 ± 1.53 a	59.26 ± 7.10 a	3.36 ± 0.16 a	0.73 ± 0.06 a	3.64 ± 0.32 a	3.26 ± 0.34 a	2.01 ± 0.31 a	2.05 ± 0.36 a
Nadir	61.88 ± 3.72 b	19.45 ± 1.53 a	11.91 ± 1.63 b	56.07 ± 8.12 b	3.22 ± 0.18 b	0.62 ± 0.07 b	3.30 ± 0.25 b	3.14 ± 0.33 b	1.82 ± 0.26 b	1.70 ± 0.33 b

*Note:* Dry matter content was assessed by calculating the percentage of dry weight in the shoots, leaves, and roots. Root colonization was quantified by determining the percentage of roots that were colonized. The concentrations of nitrogen (N), phosphorus (P), and potassium (K) were calculated as a percentage of the dry weight. Chl contents were expressed in terms of fresh weight. Data is reported as means values ± SD. The significance (*p* ≤ 0.05) among different treatments is expressed by different letters (*n* = 5).

Abbreviations: Chl, chlorophyll; K, potassium; N, nitrogen; P, phosphorus.

### 3.2 Analysis of Fruit Quality Parameters

According to Table 3, the tomato fruit quality attributes were significantly (*p* ≤ 0.05) influenced by the AMF species. The diameter (52.55 mm), firmness (3.82 kg cm^−2^), juice content (3.50%), TA (0.53%), and ascorbic acid contents (25.55%) of tomato fruit were significantly enhanced by *Glomus mosseae*. The maximum increase was recorded for *Glomus mosseae*, followed by *Glomus fasciculatum*, whereas these parameters had the lowest values in control plants (Table 5).

**TABLE 5:** Effect of AMF species on tomato fruit quality parameters (Mean ± SD).

AMF species	Fruit diameter (mm)	Fruit firmness (kg cm^−2^)	Juice content (%)	TA (%)	Ascorbic acid (%)
Control	43.01 ± 2.10e	2.63 ± 0.48e	22.45 ± 1.66e	0.43 ± 0.01e	18.22 ± 1.23e
*Glomus Claroideum*	48.10 ± 2.27d	2.87 ± 0.17d	25.93 ± 1.90d	0.48 ± 0.03d	23.09 ± 0.98d
*Glomus Etunicatum*	49.22 ± 2.36c	3.53 ± 0.19c	27.60 ± 1.87c	0.50 ± 0.02c	23.77 ± 1.13c
*Glomus Fasciculatum*	50.41 ± 2.41b	3.73 ± 0.08b	29.24 ± 1.53b	0.51 ± 0.01b	24.41 ± 1.16b
*Glomus Mosseae*	52.55 ± 2.53a	3.82 ± 0.07a	30.50 ± 1.60a	0.53 ± 0.02a	25.55 ± 1.38a

*Note:* Data are presented as the means ± SD. Significant differences (*p* ≤ 0.05) among various treatments are denoted by different letters (*n* = 10).

Abbreviations: Control, without AMF inoculation; TA, titratable acidity.

The fruit quality attributes were affected significantly (*p* ≤ 0.05) by the NP levels as well. The plants were treated with 140:42 mg L^−1^ exhibited the maximum increase in fruit size, firmness, juice content, TA, and ascorbic acid. Meanwhile, the plants treated with 100:30 mg L^−1^ showed the lowest values for fruit size, diameter, juice content, TA, and ascorbic acid (Table 6).

**TABLE 6:** Effect of NP levels on the fruit quality parameters of tomato (Mean ± SD).

AMF species	Fruit diameter (mm)	Fruit firmness (kg cm^−2^)	Juice content (%)	TA (%)	Ascorbic acid (%)
200:60 mg L^−1^	48.85 ± 2.11c	3.25 ± 0.41c	26.13 ± 1.75c	0.48 ± 0.03c	22.03 ± 1.95c
140:42 mg L^−1^	51.67 ± 2.43a	3.41 ± 0.44a	28.22 ± 1.55a	0.51 ± 0.04a	24.07 ± 2.10a
100:30 mg L^−1^	50.09 ± 2.38b	3.30 ± 0.42b	27.09 ± 1.58b	0.49 ± 0.03b	23.09 ± 1.81b

*Note:* The data is displayed as the means ± standard deviation. Significantly different treatments (*p* ≤ 0.05) are indicated by different letters (*n* = 10), as determined by ANOVA and confirmed through the Tukey test.

Abbreviation: TA, titratable acidity.

The cultivars significantly affected fruit diameter, juice content, and TA, whereas fruit firmness and ascorbic acid were not significant. The cultivar Nadir exhibited the highest values for fruit size (51.29 mm), juice content (28.48%), and TA (0.50%), whereas the lowest values were observed in the plant of the cultivar Rio Grande (Table 7).

**TABLE 7:** Effect of cultivars on the fruit quality parameters of tomato (Mean ± SD).

Cultivars	Fruit diameter (mm)	Fruit firmness (kg cm^−2^)	Juice content (%)	TA (%)	Ascorbic acid (%)
Rio grande	50.12 ± 2.31b	NS	25.82 ± 1.47b	0.48 ± 0.04b	NS
Nadir	51.29 ± 2.35a	NS	28.48 ± 1.51a	0.50 ± 0.03a	NS

*Note:* The data is presented as means ± SD. Treatments that are significantly different (*p* ≤ 0.05, *n* = 10) are denoted by distinct letters, as determined by ANOVA and validated by the Tukey test.

Abbreviation: TA, titratable acidity.

### 3.3 Interactive Effect on Growth, Physiology, Photosynthetic Pigments, and Yield of Tomato

**FIGURE 2:**
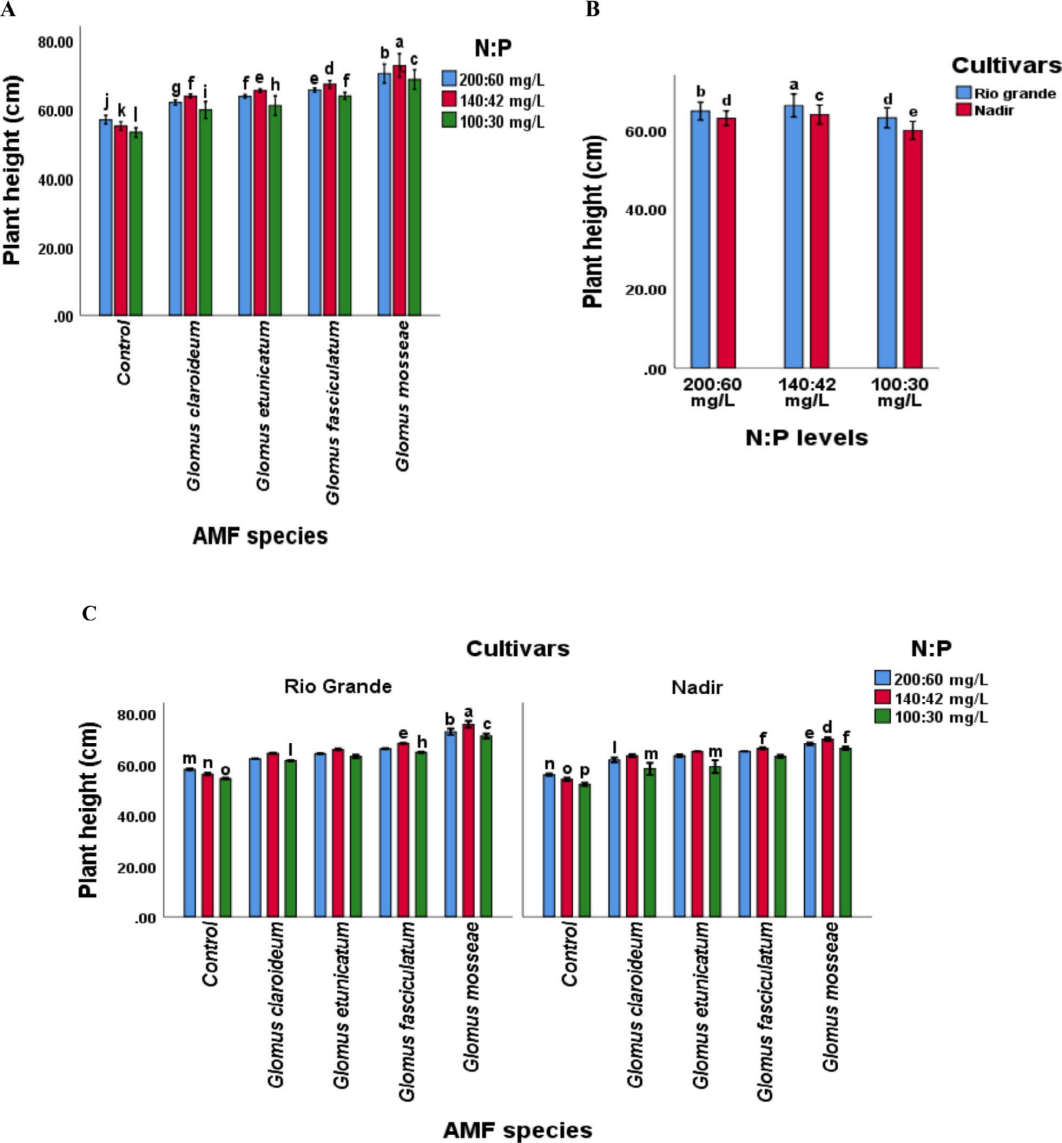
Effect of (A) AMF inoculation and NP levels on tomato height (B) Cultivars and NP levels on tomato height (C) AMF inoculation, NP levels, and cultivars on plant height. The significant difference among plant height values is represented by the different letters (*p* ≤ 0.05). AMF, arbuscular mycorrhizal fungi; Control, without AMF inoculation; NP, nitrogen and phosphorus.

**FIGURE 3:**
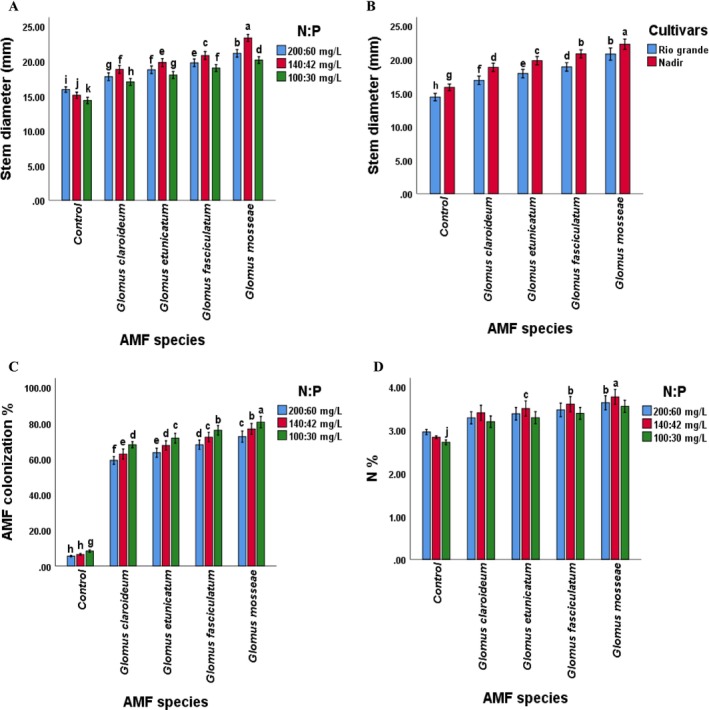
Effect of (A) AMF inoculation and NP levels on stem diameter, (B) Cultivars and AMF inoculation on stem diameter, (C) AMF inoculation and NP levels on AMF colonization, (D) AMF inoculation and NP levels on nitrogen concentration. The significance among the mean values of stem diameter is denoted by the different letters (*p* ≤ 0.05). AMF colonization and N content; AMF, arbuscular mycorrhizal fungi; Control, without AMF inoculation; NP, nitrogen and phosphorus.

**FIGURE 4:**
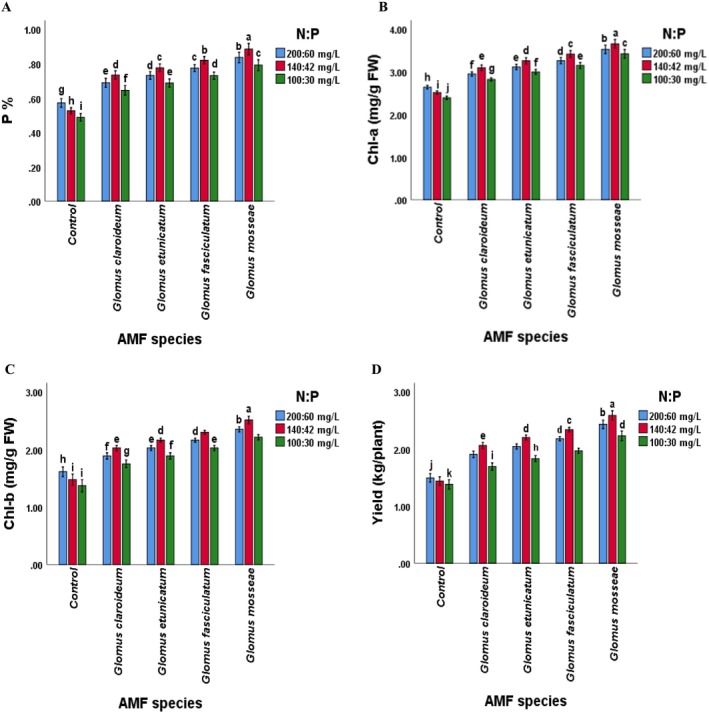
Effect of (A) AMF inoculation and NP levels on plant phosphorus concentration, (B) AMF inoculation and N:P levels on chlorophyll a content, (C) AMF inoculation and N:P levels on chlorophyll b content, (D) AMF inoculation and NP levels on yield. Different letters among the mean values of P, Chl a, Chl b, and yield indicate significant differences (*p* ≤ 0.05). AMF, arbuscular mycorrhizal fungi; Chl, chlorophyll; Control, without AMF inoculation; NP, nitrogen and phosphorus.

Considering interaction, the plant height, dry matter content, stem diameter, N, P, K, Chl contents, AMF colonization, and yield of both tomato cultivars were significantly (*p* ≤ 0.05) affected by AMF species in combination with the levels of N and P. The “GM × 140:42” treatment showed the maximum plant height, dry matter, stem diameter, N, P, K, Chl contents, and yield, whereas the “GM × 100:30” showed lower. Plant physiochemical attributes and yield were lowest in the control treatment. Overall, the values of all the observed parameters were highest in the plants treated with a combination of *Glomus mossea* and N:P levels. The other species showed a comparatively lower response but was significantly higher than the non‐inoculated. This recommends that each of the selected AMF species improved the performance of both tomato cultivars, but their percent effect was different. Maximum root inoculation was noted in plants inoculated with G. *mosseae* at 100:30 mg L^−1^ of N:P, whereas minimum root colonization was noted in non‐inoculated plants treated with 200:60 mg L^−1^ of N:P. Furthermore, the maximum plant height was found in plants of Rio Grande treated with 140:42 mg L^−1^ of NP, whereas the minimum was shown in the Nadir cultivar treated with 100:30 mg L^−1^ of N:P, suggesting that the interaction of cultivars and NP levels significantly affected plant height. Nadir plants treated with 140:42 mg L^−1^ of NP showed the maximum stem diameter, whereas the minimum was observed in plants of Rio Grande treated with 100:30 mg L^−1^ of N:P. The combination of *Glomus mossea* and NP level of 140:42 mg L^−1^ and cultivar Rio Grande recorded the highest plant height while the lowest was noted in non‐inoculated plants of Nadir treated with 100:30 mg L^−1^ of NP. Compared to other species, the values of all of the studied parameters were found to be highest in the plants treated with a combination of *Glomus mossea* and NP levels followed by *Glomus fasciculatum*. In terms of growth, physiological characteristics, and yield, the *Glomus etunicatum* was ranked third. However, all the studied AMF species showed improvement in the values of plant height, dry matter, stem diameter, NPK, Chl contents, AMF colonization and yield, compared to non‐inoculated tomato plants (Figures 2, 3 and 4).

### 3.4 Interactive Effects on Fruit Quality

**FIGURE 5:**
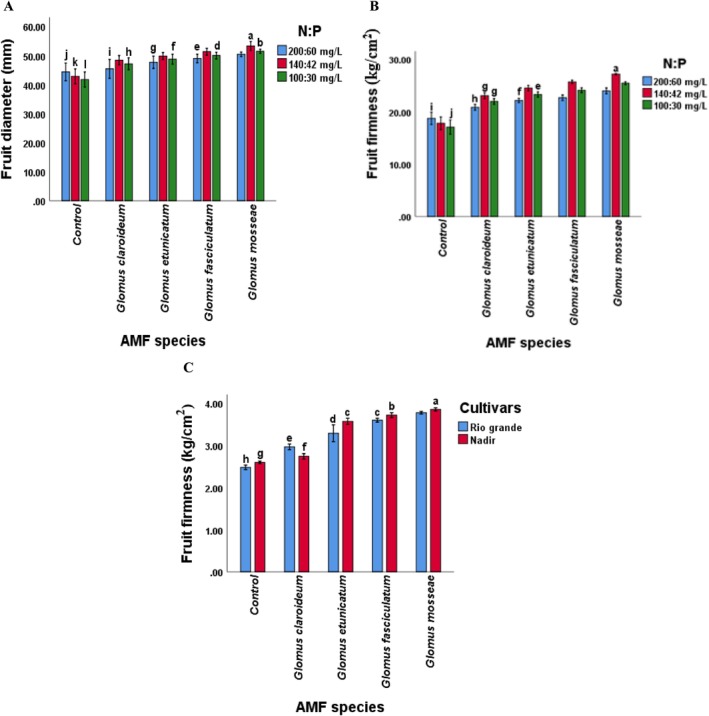
Effect of (A) AMF inoculation and NP levels on the fruit diameter (B) AMF inoculation and NP levels on stem diameter (C) AMF inoculation and cultivars on stem diameter of tomato. Different letters among fruit diameter and firmness values indicate significant differences (*p* ≤ 0.05). Fruit diameter has been noted in mm. Fruit firmness has been shown in kg cm^−2^. AMF, arbuscular mycorrhizal fungi; Chl, chlorophyll; Control, without AMF inoculation; NP, nitrogen and phosphorus.

**FIGURE 6:**
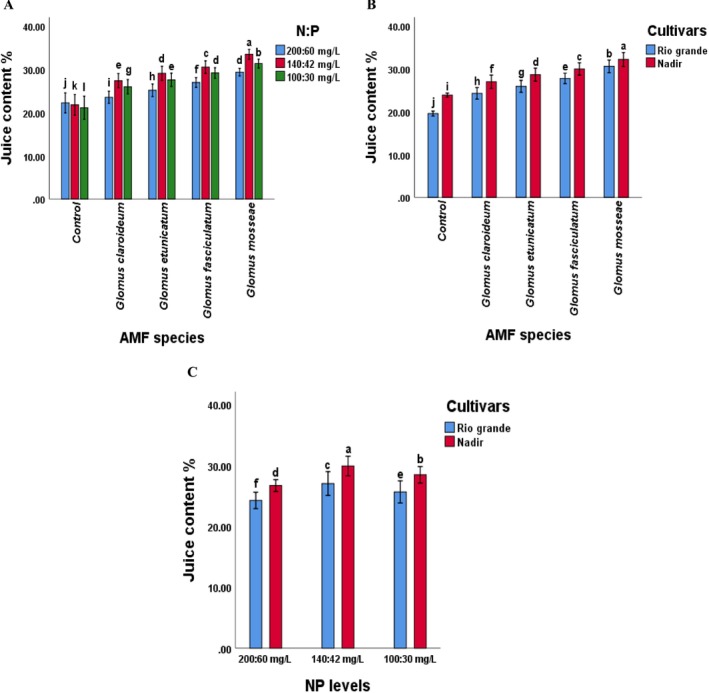
Effect of (A) AMF inoculation and NP levels on juice content (B) AMF inoculation and cultivars on juice content (C) NP levels and cultivars of tomato. The different letters among the values of fruit juice content indicate significant differences (*p* ≤ 0.05). AMF, arbuscular mycorrhizal fungi; Chl, chlorophyll; Control, without AMF‐inoculation; NP, nitrogen and phosphorus.

**FIGURE 7:**
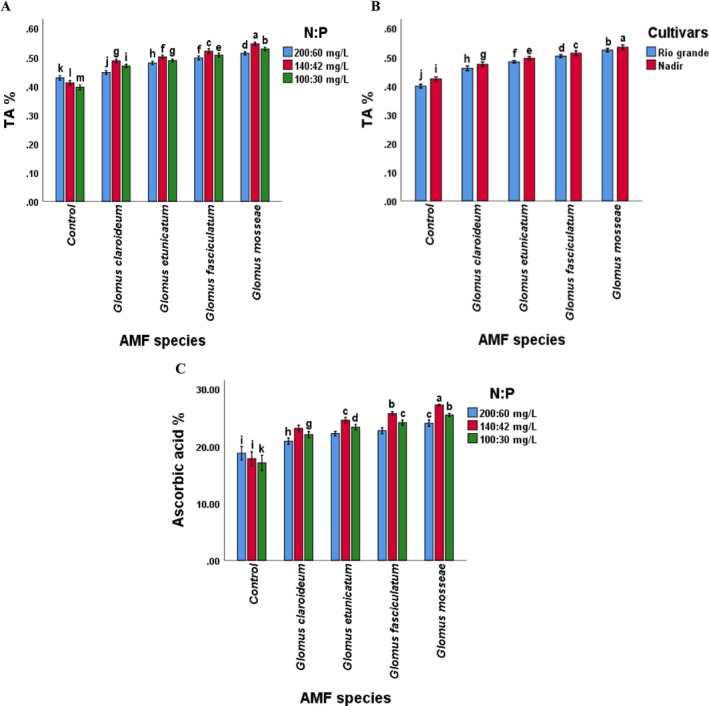
Effect of (A) AMF inoculation and NP levels on TA (B) AMF inoculation and cultivars on TA (C) AMF inoculation and NP levels on ascorbic acid content of tomato. Different letters among the mean values of titratable acidity and ascorbic acid contents indicate significant differences (*p* ≤ 0.05). AMF, arbuscular mycorrhizal fungi; Chl, chlorophyll; Control, without AMF inoculation; NP, nitrogen and phosphorus.

Regarding the interaction, the diameter, firmness, juice content, ascorbic acid content, and TA of tomato fruit was affected by the combination of AMF species and NP levels (*p* < 0.05). The treatment “GM × 140:42” demonstrated the highest recorded values for fruit diameter, firmness, juice content, TA, and ascorbic acid content (Figures 5A,B, 6A, 7A,C). The fruit quality attributes were the lowest in the control treatment. When compared to other treatments, the plants treated with *Glomus mossea* and NP levels exhibited the highest values for all parameters. The *Glomus fasciculatum* ranked second in performance concerning fruit quality parameters. Although the other species showed a comparatively lower response but this response was significantly higher as compared to the control. This indicates that each of the selected AMF species improved the performance of tomato cultivars.

Furthermore, the maximum increase in fruit firmness, juice content, and TA content was noted in fruits of cultivar Nadir inoculated with G *mossea*, whereas the minimum was recorded in the fruits of Rio Grande in control plants (Figures 5C, 6B and 7B), suggesting that the interaction of AMF and cultivars significantly affected the fruit firmness, juice content, and TA content of tomato fruits. Additionally, the interaction of NP levels and cultivars significantly influenced the juice content (Figure 6C).

When compared to other species, the values of all of the studied parameters were found to be highest in the fruits of those plants that were treated with a combination of *Glomus mossea* and NP levels, followed by *Glomus fasciculatum*. In terms of fruit quality attributes, *Glomus tunicate* was ranked third; however, all the AMF species showed higher values of the fruit quality parameters than non‐inoculated plants.

### 3.5 Correlation Analysis for the Physiological, Yield, and Quality Parameters

**FIGURE 8:**
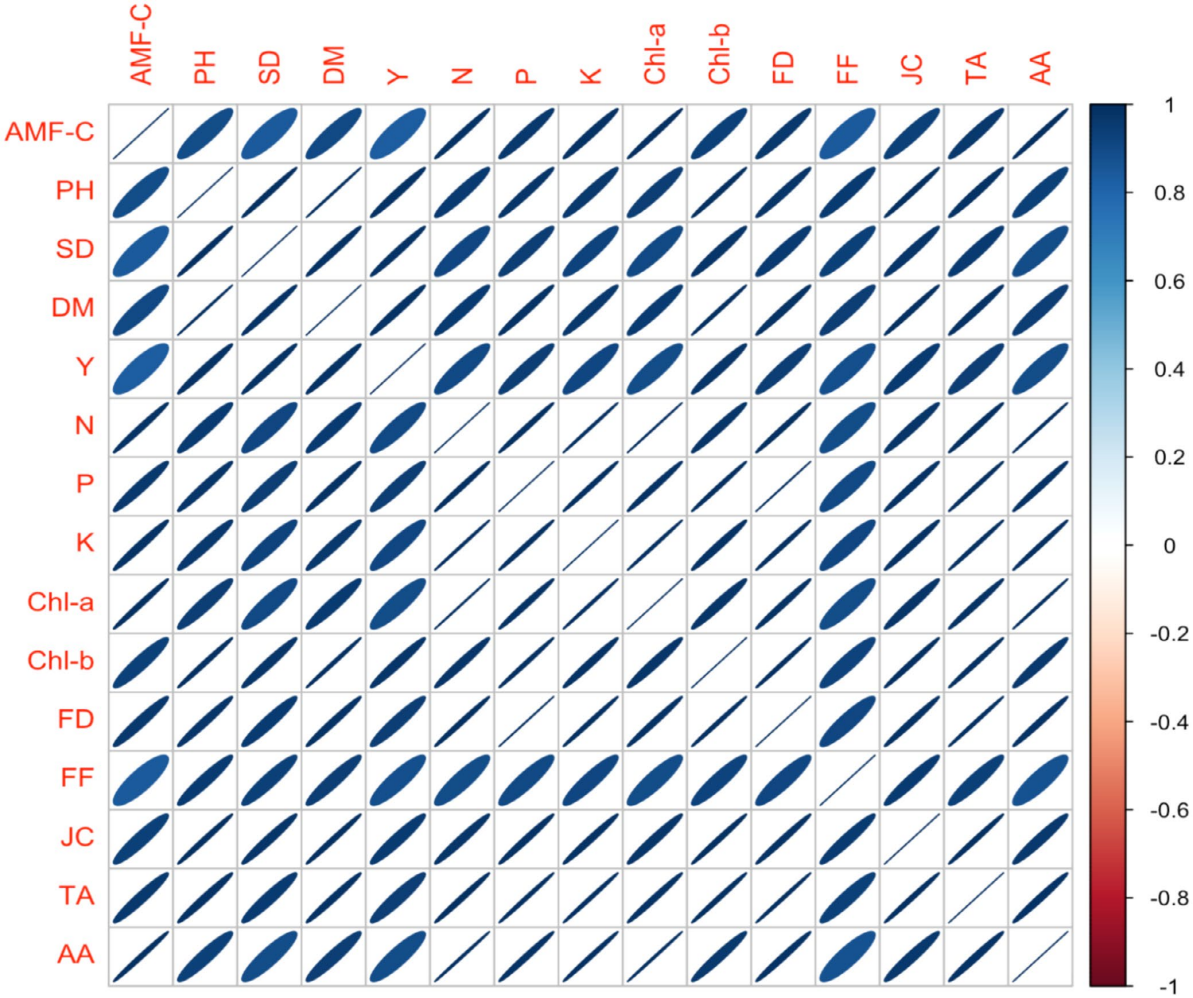
Correlations of the physiological, production, and quality parameters of Rio Grande and Nadir cultivars affected by AMF species. AA, ascorbic acid; AMF‐C, without AMF inoculation; Chl a, chlorophyll a; Chl b, chlorophyll b; DM, dry matter; FD, fruit diameter; FF, fruit firmness; JC, juice content; K, potassium; N, nitrogen; P, phosphorus; PH, plant height; SD, stem diameter; TA, titratable acidity; Y, yield.

The detailed analysis of the scattered plot matrix, shown in Figure 8, provided convincing evidence of strong positive correlations among the observed variables. This visual representation described the strong interconnections existing between these variables, showing a clear trend where changes in one variable were consistently accompanied by corresponding changes in the others. The presence of these positive correlations emphasizes the coherent and interdependent nature of the variables under consideration. It is shedding light on the complex relationships within the dataset and enhancing our understanding of the underlying patterns and interactions among the observed variables. This comprehensively overviews how different growth parameters, nutrient contents, and fruit quality attributes were interrelated. The strength and direction of each correlation indicate that an increase in root colonization led to an increase in N and P uptake, resulting in a higher stem diameter and plant height. According to Figure 8, the increase in these nutrients' translocation also led to an increase in Chl contents, carotenoid contents, and yield. The values of fruit quality parameters increased with the increase in photosynthetic pigments, P accumulation, and root colonization. The colonization of AMF resulted in enhanced plant growth, increased yield, and improved fruit quality. This detailed correlation analysis clearly shows the importance of proper nutrient intake, especially N and P, for tomato plants to grow vigorously and produce high‐quality fruits. Increased root colonization played a significant role in facilitating the uptake of these nutrients.

### 3.6 Principal Component Analysis (PCA) of Observed Variables

**FIGURE 9:**
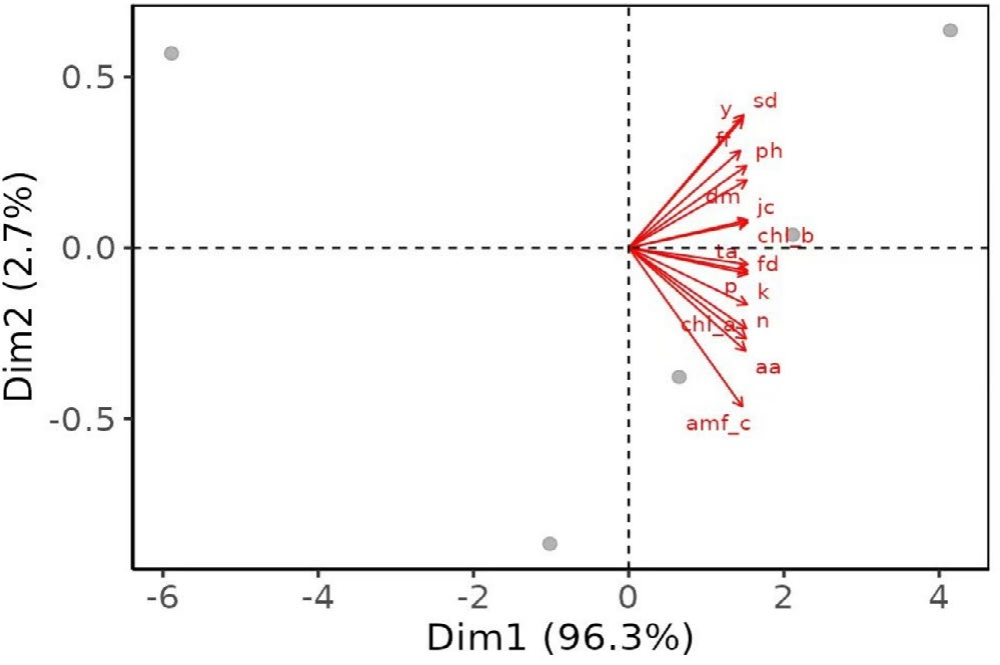
Principal component analysis (PCA) of different observed variables. aa, ascorbic acid; amf‐C, without AMF inoculation; chl a, chlorophyll a; chl b, chlorophyll b; dm, dry matter; fd, fruit diameter; ff, fruit firmness; jc, juice content; k, potassium; n, nitrogen; p, phosphorus; phpH, plant height; sd, stem diameter; ta, titratable acidity; y, yield.

Principal component analysis (PCA) was performed to investigate the multivariate relationships among observed variables. The first two principal components (Dim1 and Dim2) explained 96.3% and 2.7% of the total variance, respectively, accounting for a cumulative variance of 99% (Figure 9). Dim1 dominated the separation of variables, indicating that the majority of variation in the dataset was captured along this axis. Most variables, including soil nutrients (e.g., N, P, K), physicochemical properties (e.g., pH, sd, fd), and other indicators (e.g., chla, chlb, aa, amf_c), were strongly aligned in the positive direction of Dim1, suggesting strong positive correlations among them. The consistent orientation of the vectors along Dim1 reflects a high degree of shared variance, whereas the minor contribution of Dim2 suggests limited differentiation along this axis.

## 4 Discussion

Our comprehensive study demonstrates that AMF significantly enhance tomato growth and nutrient uptake through multifaceted mechanisms. The superior performance of *Glomus mosseae* in promoting plant height, dry matter, stem diameter, and NPK uptake (Tables 2, 3, 4) aligns with previous reports of AMF benefits in tomatoes (Douds et al. 2007; Salvioli et al. 2012). Ullah, Ullah, Ishfaq, Gul, et al. (2023), Ullah, Ullah, Ishfaq, Khan, et al. (2023). AMF produce extraradical hyphae (tiny fungal filaments) that extend far beyond the root zone. These hyphae can access nutrients from soil areas that roots alone can't reach, especially important for immobile nutrients like phosphorus (P), zinc (Zn), and copper (Cu) (Smith and Smith 2011). AMF are very efficient at absorbing and transferring phosphates to the host plant. Some AMF can even release organic acids and enzymes that help solubilize P locked in soil particles, making it available for the plant. Tomato plants get more P, essential for energy (ATP) and growth. Fungal hyphae also help absorb water more efficiently. AMF colonization often modulates plant hormones like auxins, cytokinins, and abscisic acid (ABA) (Prasad 2021). These hormones can stimulate root growth (more root hairs, branching), enhancing the plant's own ability to absorb minerals. AMF also help aggregate soil particles by producing a sticky substance called glomalin. Better soil structure improves aeration, water retention, and nutrient holding capacity, increasing nutrients available in the root zone. This enhanced growth directly correlates with increased root colonization rates (Figure 8), supporting Berta et al.'s (2005) findings on AMF‐mediated root development. The symbiotic relationship facilitates nutrient exchange through sophisticated mechanisms—inorganic P (Pi) transport as polyphosphate (Funamoto et al. 2007) and N as arginine‐bound polyphosphate (Tian et al. 2010; Jin et al. 2005), with fungal gene expression tightly coupled to host carbon supply (Fellbaum et al. 2012).

The critical interaction between AMF efficacy and NP levels reveals important trade‐offs. Although maximum colonization occurred at 100:30 mg L^−1^ NP, optimal growth and yield required 140:42 mg L^−1^ (Table 3), confirming that moderate fertilization sustains both AMF symbiosis and productivity (Breuillin et al. 2010; Schubert et al. 2020). This supports Ortas' (2012) proposal for partial fertilizer substitution, with our data showing AMF can reduce inputs by 50% (Begum et al. 2019) while maintaining yield. However, high P levels (200:60 mg L^−1^) suppressed colonization, consistent with Balzergue et al.'s (2011) findings on P‐regulated colonization. Paradoxically, low‐N conditions sometimes stimulated mycorrhization (Olsson et al. 2005; Bunn et al. 2024), highlighting the complex nutrient–AMF interplay that requires cultivar‐specific optimization (Pellegrino et al. 2011).

Physiologically, AMF colonization dramatically improved photosynthetic efficiency through increased chlorophyll content (Delavaux et al. 2017; Gupta and Seth 2021; Yadav et al. 2022), particularly in P‐limited plants (Ishfaq et al. 2023). This enhanced photosynthesis promotes photoassimilate production (Candido et al. 2013), driving the observed growth improvements. The cultivar‐specific responses—Rio Grande's superior growth versus Nadir's better fruit quality (Tables 4 and 7)—suggest genetic factors modulate AMF effects, possibly through differential carbon allocation (Zaheer, Aijaz, et al. 2024; Zaheer, Ali, et al. 2024). These findings expand on Wakeel and Ishfaq's (2016, 2022) work on AMF‐mediated nutrient availability in tomatoes. Some AMF species are better at P uptake (e.g., Rhizophagus irregularis), whereas others are more efficient with N. At low P levels, AMF upregulate P transporters and enhance P acquisition for the host plant. AMF can improve organic and inorganic N absorption through hyphae, supplying amino acids or ammonium to plants. Under varying N and P, AMF alter plant root systems differently. For example, some AMF induce more lateral roots under P deficiency, improving P scavenging (Chaudhary et al. 2025). Others stimulate fine root development for better N uptake under N‐limiting conditions. AMF modulate key enzymes depending on N and P levels: phosphatases, e.g., acid phosphatase activity increases under P deficiency to release P from organic compounds. Nitrate reductase and glutamine synthetase activities are enhanced under low N to help assimilate available N (Sardans et al. 2023). Adequate N and P through AMF inoculation boost photosynthetic rates, since N is needed for chlorophyll, and P for ATP/energy. Some AMF species cause higher carbon allocation to roots and fungal partners under low nutrient conditions to sustain the symbiosis. AMF colonization under nutrient stress changes levels of auxins, cytokinins, and abscisic acid (ABA). Different AMF may enhance auxin under low P (to stimulate root growth) or increase ABA under low N to reduce stress.

Remarkably, AMF inoculation significantly enhanced multiple fruit quality parameters (Tables 5, 6), increasing beneficial compounds like ascorbic acid and carotenoids while reducing undesirable elements (Bhantana et al. 2021; Ait‐El‐Mokhtar et al. 2020). The treatment “GM × 140:42” showed optimal results, demonstrating that balanced nutrition with AMF maximizes quality. These improvements stem from AMF‐induced metabolic changes, including elevated antioxidant enzyme activity (APX, CAT, SOD) that enhances shelf life (Shalaby and Ramadan 2024). The advanced flowering and fruit maturation (Schubert et al. 2020) further confirm AMF's role in modifying developmental timing. Although hydroponic systems typically suppress AMF (Hart et al. 2015; Bona et al. 2018), our findings show that moderate P reduction maintains functional symbiosis (Schubert et al. 2020), supporting AMF use in diverse production systems. The hyphal network releases carbon compounds (exudates) into the soil, providing food sources that stimulate microbial growth. AMF can favor the growth of specific groups, like plant growth‐promoting rhizobacteria (PGPR) and nitrogen‐fixing bacteria. These beneficial microbes often work synergistically with AMF, improving plant nutrition and health. By occupying root space and competing for resources, AMF can also limit the growth of pathogenic microbes (Boutaj et al. 2025). Some AMF also induce plant defenses, indirectly reducing pathogen populations in the rhizosphere. AMF modifies the chemical environment around the roots e.g., pH, nutrient availability. This chemical shift can change which microbial species thrive or decline (Muhammad et al. 2025).

**FIGURE 10:**
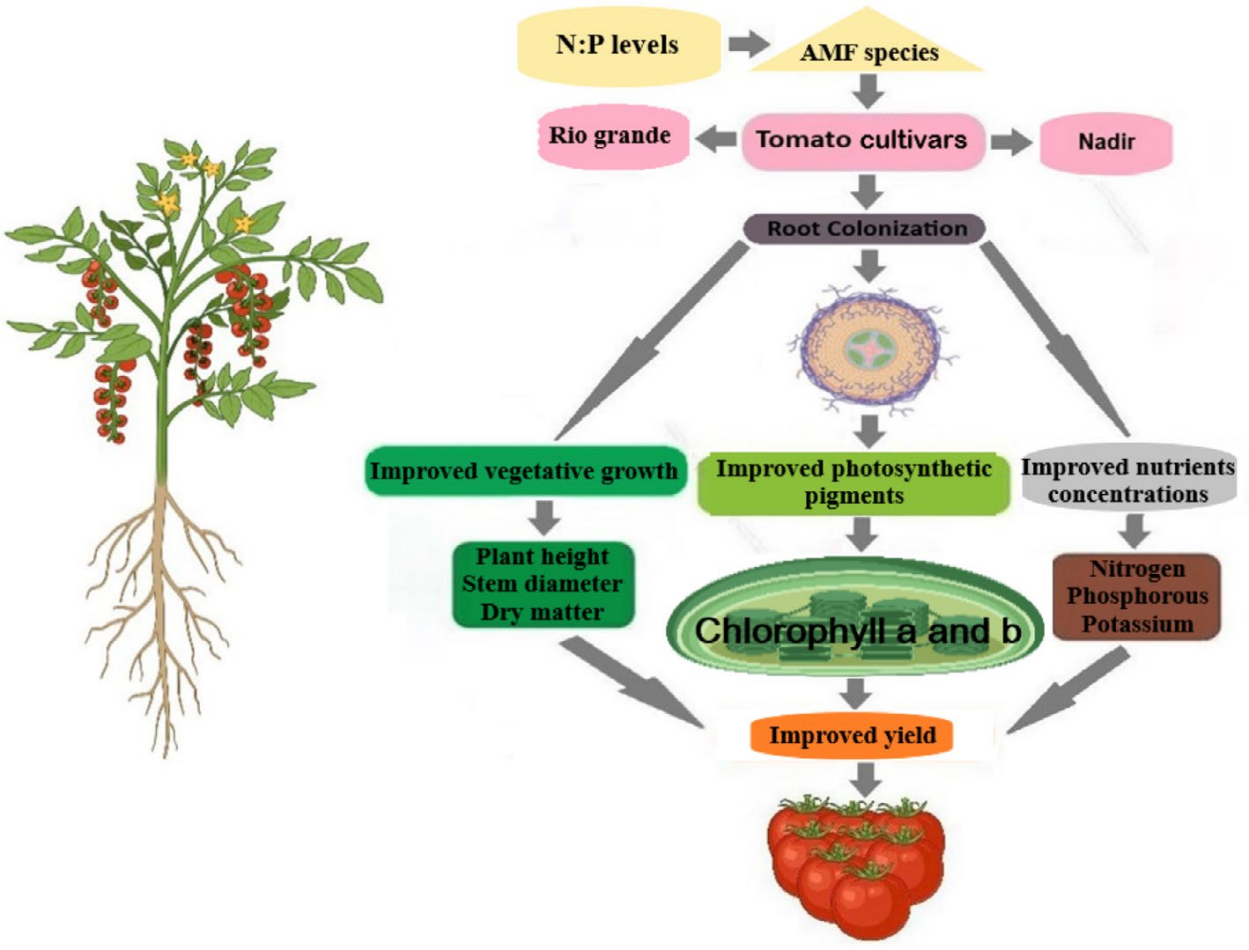
Schematic illustration of AMF inoculation in promoting plant growth, nutrient uptake, photosynthetic activity, and yield of tomato. AMF, arbuscular mycorrhizal fungi; NP, nitrogen and phosphorus.

These results have profound implications for sustainable agriculture. By combining AMF with optimized fertilization (Bi et al. 2018), growers can reduce chemical inputs while enhancing both yield and fruit quality. The strong correlations between colonization, nutrient uptake, and quality parameters validate AMF's integral role in tomato production systems (Figure 10). Future research should explore field applications (Salvioli et al. 2012) and investigate soil microbiome interactions to fully realize AMF's potential in sustainable horticulture.

## 5 Conclusions

Our study demonstrates that AMF inoculation, particularly with *Glomus mosseae*, significantly enhances tomato performance by improving nutrient uptake (NPK concentrations increased by 30%–50%) and photosynthetic activity (higher chlorophyll a/b), especially when combined with moderate NP fertilization (140:42 mg L^−1^). *G. mosseae* outperformed other species, boosting growth and yield, whereas cultivar “Rio Grande” showed greater responsiveness than “Nadir,” likely due to efficient AMF‐mediated nutrient allocation. These findings propose a sustainable strategy: reducing chemical fertilizers by 30%–50% without compromising productivity, as AMF sustains yield and enhances fruit quality (e.g., ascorbic acid, firmness). Future research should validate these results in field conditions, explore cultivar‐specific AMF interactions, and investigate soil microbiome dynamics to optimize scalable, eco‐friendly tomato production. This approach paves the way for reducing agricultural chemical dependency while addressing food security challenges.

## Ethics Statement

The authors have nothing to report.

## Consent

The authors have nothing to report.

## Conflicts of Interest

The authors declare no conflicts of interest.

## Data Availability

The data that support the findings of this study are available on request from the corresponding author. The data are not publicly available due to privacy or ethical restrictions.

## References

[pei370058-bib-0001] Ahmed, N. , J. Li , Y. Li , et al. 2025. “Symbiotic Synergy: How Arbuscular Mycorrhizal Fungi Enhance Nutrient Uptake, Stress Tolerance, and Soil Health Through Molecular Mechanisms and Hormonal Regulation.” IMA Fungus 16: e144989. 40162002 10.3897/imafungus.16.144989 PMC11953731

[pei370058-bib-0002] Ait‐El‐Mokhtar, M. , M. Baslam , R. Ben‐Laouane , et al. 2020. “Alleviation of Detrimental Effects of Salt Stress on Date Palm (*Phoenix dactylifera* L.) by the Application of Arbuscular Mycorrhizal Fungi and/or Compost.” Frontiers in Sustainable Food Systems 4: 131.

[pei370058-bib-0003] Alhammad, B. A. , M. S. Zaheer , H. H. Ali , A. Hameed , K. Z. Ghanem , and M. F. Seleiman . 2023. “Effect of Co‐Application of *Azospirillum brasilense* and *Rhizobium pisi* on Wheat Performance and Soil Nutrient Status Under Deficit and Partial Root Drying Stress.” Plants 12: 3141. 37687389 10.3390/plants12173141 PMC10489886

[pei370058-bib-0004] Ali, H. H. , N. Shehzadi , M. S. Zaheer , et al. 2023. “Exploring the Impact of Salicylic Acid and Farmyard Manure on Soil Rhizospheric Properties and Cadmium Stress Alleviation in Maize (*Zea mays* L.).” Plants 17: 3115. 10.3390/plants12173115 PMC10490018 37687361

[pei370058-bib-0005] AOAC . 1980. Official Methods of Analysis, 13th Edition. Association of Official Agricultural Chemists.

[pei370058-bib-0006] AOAC . 1990. Official Methods of Analysis of the Association of Official Analytical Chemists, Vol. II, 15th edn. Sec. 985.29. Association.

[pei370058-bib-0007] Arendse, E. , H. Nieuwoudt , O. A. Fawole , and U. L. Opara . 2021. “Effect of Different Extraction Methods on the Quality and Biochemical Attributes of Pomegranate Juice and the Application of Fourier Transformed Infrared Spectroscopy in Discriminating Between Different Extraction Methods.” Frontiers in Plant Science 12: 702575. 10.3389/fpls.2021.702575. 34497620 PMC8419332

[pei370058-bib-0009] Balzergue, C. , V. Puech‐Pagès , G. Bécard , and S. F. Rochange . 2011. “The Regulation of Arbuscular Mycorrhizal Symbiosis by Phosphate in Pea Involves Early and Systemic Signaling Events.” Journal of Experimental Botany 62: 1049–1060. 10.1093/jxb/erq335. 21045005 PMC3022399

[pei370058-bib-0010] Begum, N. , C. Qin , M. A. Ahanger , et al. 2019. “Role of Arbuscular Mycorrhizal Fungi in Plant Growth Regulation: Implications in Abiotic Stress Tolerance.” Frontiers in Plant Science 10: 1068. 10.3389/fpls.2019.01068. 31608075 PMC6761482

[pei370058-bib-0011] Behera, K. K. , S. Mohanty , U. Kumar , M. Kaviraj , and R. Padbhushan . 2024. Microbiome and Plant Nutrition: A Roadmap Towards Sustainable Agriculture. CRC Press.

[pei370058-bib-0012] Berta, G. , S. Sampo , E. Gamalero , N. Massa , and P. Lemanceau . 2005. “Suppression of Rhizoctonia Root‐Rot of Tomato by Glomus Mossae BEG12 and *Pseudomonas fluorescens* A6RI Is Associated With Their Effect on the Pathogen Growth and on the Root Morphogenesis.” European Journal of Plant Pathology 111: 279–288. 10.1007/s10658-005-3129-1.

[pei370058-bib-0013] Bhantana, P. , M. S. Rana , X. C. Sun , et al. 2021. “Arbuscular Mycorrhizal Fungi and Its Major Role in Plant Growth, Zinc Nutrition, Phosphorous Regulation and Phytoremediation.” Symbiosis 84: 19–37. 10.1007/s13199-021-00765-7.

[pei370058-bib-0014] Bi, Y. , L. Qiu , Y. Zhakypbek , B. Jiang , Y. Cai , and H. Sun . 2018. “Combination of Plastic Film Mulching and AMF Inoculation Promotes Maize Growth, Yield and Water Use Efficiency in the Semiarid Region of Northwest China.” Agricultural Water Management 201: 287–296. 10.1016/j.agwat.2017.12.008.

[pei370058-bib-0015] Bona, E. , V. Tardivo , S. Cantamessa , et al. 2018. “Combined Bacterial and Mycorrhizal Inocula Improve Tomato Quality at Reduced Fertilization.” Scientia Horticulturae 234: 160–165. 10.1016/j.scienta.2018.02.026.

[pei370058-bib-0017] Boutaj, H. , A. Meddich , S. Wahbi , et al. 2025. “Mycorrhizal Fungus Rhizophagus Irregularis Mitigates Symptoms of Verticillium Wilt and Enhances Growth of Olive Trees (*Olea europaea* L.).” European Journal of Plant Pathology 172: 1–17.

[pei370058-bib-0018] Breuillin, F. , J. Schramm , M. Hajirezaei , et al. 2010. “Phosphate Systemically Inhibits Development of Arbuscular Mycorrhiza in *Petunia hybrida* and Represses Genes Involved in Mycorrhizal Functioning.” Plant Journal 64, no. 6: 1002–1017. 10.1111/j.1365-313X.2010.04387.x. 21143680

[pei370058-bib-0019] Bunn, R. A. , A. Corrêa , J. Joshi , et al. 2024. “What Determines Transfer of Carbon From Plants to Mycorrhizal Fungi?” New Phytologist 244, no. 4: 1199–1215. 39352455 10.1111/nph.20145

[pei370058-bib-0020] Candido, V. , G. Campanelli , T. D'Addabbo , D. Castronuovo , M. Renco , and I. Camele . 2013. “Growth and Yield Promoting Effect of Artificial Mycorrhization Combined With Different Fertiliser Rates on Field‐Grown Tomato.” Italian Journal of Agronomy 8: e22. 10.4081/ija.2013.e22.

[pei370058-bib-0021] Casieri, L. , K. Gallardo , and D. Wipf . 2012. “Transcriptional Response of *Medicago truncatula* Sulfate Transporters to Arbuscular Mycorrhizal Symbiosis With and Without Sulfur Stress.” Planta 235, no. 6: 1431–1447. 22535379 10.1007/s00425-012-1645-7

[pei370058-bib-0022] Chaudhary, A. , S. Poudyal , and A. Kaundal . 2025. “Role of Arbuscular Mycorrhizal Fungi in Maintaining Sustainable Agroecosystems.” Applied Microbiology 5, no. 6: 1.

[pei370058-bib-0023] Che, X. , S. Wang , Y. Ren , et al. 2022. “A Eucalyptus Pht1 Family Gene EgPT8 Is Essential for Arbuscule Elongation of *Rhizophagus irregularis* .” Microbiology Spectrum 10, no. 6: e01470‐22. 36227088 10.1128/spectrum.01470-22 PMC9769952

[pei370058-bib-0024] Cheaib, A. , J. Chieppa , E. A. Perkowski , and N. G. Smith . 2025. “Soil Resource Acquisition Strategy Modulates Global Plant Nutrient and Water Economics.” New Phytologist 246: 1536–1553. 10.1111/nph.70087. 40123121

[pei370058-bib-0025] Cornea‐Cipcigan, M. , A. Bunea , C. M. Bouari , et al. 2022. “Anthocyanins and Carotenoids Characterization in Flowers and Leaves of Cyclamen Genotypes Linked With Bioactivities Using Multivariate Analysis Techniques.” Antioxidants 11, no. 6: 1126. 10.3390/antiox11061126. 35740023 PMC9220265

[pei370058-bib-0026] Delavaux, C. S. , L. M. Smith‐Ramesh , and S. E. Kuebbing . 2017. “Beyond Nutrients: A Meta‐Analysis of the Diverse Effects of Arbuscular Mycorrhizal Fungi on Plants and Soils.” Ecology 98, no. 8: 2111–2119. 10.1002/ecy.1892. 28500779

[pei370058-bib-0027] Douds, D. D. , G. Nagahashi , C. Reider , and P. R. Hepperly . 2007. “Inoculation With Arbuscular Mycorrhizal Fungi Increases the Yield of Potatoes in a High P Soil.” Biological Agriculture & Horticulture 25, no. 1: 67–78. 10.1080/01448765.2007.10823209.

[pei370058-bib-0028] Fellbaum, C. R. , E. W. Gachomo , Y. Beesetty , et al. 2012. “Carbon Availability Triggers Fungal Nitrogen Uptake and Transport in Arbuscular Mycorrhizal Symbiosis.” Proceedings of the National Academy of Sciences of the United States of America 14, no. 6: 2666–2671. 10.1073/pnas.1118650109. PMC3289346 22308426

[pei370058-bib-0029] Franco, M. , and M. Rinne . 2023. “Dry Matter Content and Additives With Different Modes of Action Modify the Preservation Characteristics of Grass Silage.” Fermentation 9, no. 7: 640. 10.3390/fermentation9070640.

[pei370058-bib-0030] Funamoto, R. , K. Saito , H. Oyaizu , M. Saito , and T. Aono . 2007. “Simultaneous In Situ Detection of Alkaline Phosphatase Activity and Polyphosphate in Arbuscules Within Arbuscular Mycorrhizal Roots.” Functional Plant Biology 34, no. 9: 803–810. 10.1071/FP06326. 32689408

[pei370058-bib-0031] Giovannetti, M. , and B. Mosse . 1980. “An Evaluation of Techniques for Measuring Vesicular Arbuscular Mycorrhizal Infection in Roots.” New Phytologist 84, no. 3: 489–500.

[pei370058-bib-0032] Gupta, S. , and C. S. Seth . 2021. “Salicylic Acid Alleviates Chromium (VI) Toxicity by Restricting Its Uptake, Improving Photosynthesis and Augmenting Antioxidant Defense in *Solanum lycopersicum* L.” Physiology and Molecular Biology of Plants 27: 2651–2664. 10.1007/s12298-021-01088-x. 34924716 PMC8639991

[pei370058-bib-0033] Hart, M. , D. L. Ehret , A. Krumbein , et al. 2015. “Inoculation With Arbuscular Mycorrhizal Fungi Improves the Nutritional Value of Tomatoes.” Mycorrhiza 25, no. 5: 359–376. 25391485 10.1007/s00572-014-0617-0

[pei370058-bib-0034] Hodge, A. , and K. Storer . 2015. “Arbuscular Mycorrhiza and Nitrogen: Implications for Individual Plants Through Ecosystems.” Plant and Soil 386, no. 1: 1–9.

[pei370058-bib-0035] Ishfaq, M. , A. Kiran , U. H. Rehman , et al. 2022. “Foliar Nutrition: Potential and Challenges Under Multifaceted Agriculture.” Environmental and Experimental Botany 200: 104909. 10.1016/j.envexpbot.2022.104909.

[pei370058-bib-0036] Ishfaq, M. , A. Kiran , A. Wakeel , M. Tayyab , and X. Li . 2023. “Foliar‐Applied Potassium Triggers Soil Potassium Uptake by Improving Growth and Photosynthetic Activity of Wheat and Maize.” Journal of Plant Nutrition 46, no. 11: 2691–2706. 10.1080/01904167.2022.2160748.

[pei370058-bib-0037] Ishfaq, M. , N. F. Y. Tam , T. Lang , M. Hussain , and H. Zhou . 2025. “Nitrogen‐Phosphorus Conservation and Trade‐Offs in Mangroves.” Plant and Soil. 10.1007/s11104-024-07130-7.

[pei370058-bib-0038] Ishfaq, M. , Y. Wang , M. A. Nawaz , H. Zhou , and X. Li . 2025. “Transcriptome Profiling of Tomato Fruit Ripening and Postharvest Quality Response to Magnesium Deficiency Stress.” Plant and Soil. 10.1007/s11104-025-07298-6.

[pei370058-bib-0039] Ishfaq, M. , Y. Wang , J. Xu , et al. 2023. “Improvement of Nutritional Quality of Food Crops With Fertilizer: A Global Meta‐Analysis.” Agronomy for Sustainable Development 43: 74. 10.1007/s13593-023-00923-7.

[pei370058-bib-0041] Ishfaq, M. , Y. Zhong , Y. Wang , and X. Li . 2021. “Magnesium Limitation Leads to Transcriptional Down‐Tuning of Auxin Synthesis, Transport, and Signaling in the Tomato Root.” Frontiers in Plant Science 12: 802399. 10.3389/fpls.2021.802399. 35003191 PMC8733655

[pei370058-bib-0044] Jin, H. , P. Pfeffer , D. Douds , E. Piotrowski , P. Lammers , and Y. Shachar‐Hill . 2005. “The Uptake, Metabolism, Transport and Transfer of Nitrogen in an Arbuscular Mycorrhizal Symbiosis.” New Phytologist 168: 687–696. 10.1111/j.1469-8137.2005.01536.x. 16313650

[pei370058-bib-0045] Kanwal, H. , A. Raza , M. S. Zaheer , et al. 2024. “Transformation of Heavy Metals From Contaminated Water to Soil, Fodder and Animals.” Scientific Reports 22;14(1):11705 14: 11705. 38778064 10.1038/s41598-024-62038-7 PMC11111443

[pei370058-bib-0046] Mehmood, I. , K. I. Wani , and T. Aftab . 2025. Strigolactone Interplay With Other Phytohormones Under Stressed and Normal Conditions, 161–191. Emerging Plant Hormones.

[pei370058-bib-0047] Muhammad, M. , A. Wahab , A. Waheed , et al. 2025. “Navigating Climate Change: Exploring the Dynamics Between Plant–Soil Microbiomes and Their Impact on Plant Growth and Productivity.” Global Change Biology 31, no. 2: e70057. 39924996 10.1111/gcb.70057

[pei370058-bib-0048] Olsson, P. A. , S. H. Burleigh , and I. M. Van Aarle . 2005. “The Influence of External Nitrogen on Carbon Allocation to Glomus Intraradices in Monoxenic Arbuscular Mycorrhiza.” New Phytologist 168: 677–686. 10.1111/j.1469-8137.2005.01532.x. 16313649

[pei370058-bib-0049] Ortas, I. 2012. “The Effect of Mycorrhizal Fungal Inoculation on Plant Yield, Nutrient Uptake and Inoculation Effectiveness Under Long‐Term Field Conditions.” Field Crops Research 125: 35–48. 10.1016/j.fcr.2011.08.005.

[pei370058-bib-0050] Pellegrino, E. , S. Bedini , L. Avio , E. Bonari , and M. Giovannetti . 2011. “Field Inoculation Effectiveness of Native and Exotic Arbuscular Mycorrhizal Fungi in a Mediterranean Agricultural Soil.” Soil Biology and Biochemistry 43: 367–376. 10.1016/j.soilbio.2010.11.002.

[pei370058-bib-0052] Plocharski, W. J. , D. Konopacka , and J. Zwierz . 2000. “Comparison of Magness‐Taylor's Pressure Test With Mechanical, Non‐Destructive Methods of Apple and Pear Firmness Measurements.” International Agrophysics 14: 311–318.

[pei370058-bib-0053] Prasad, K. 2021. “Glycoprotein Producing AM Fungi Lifecycle and Potential Role in Agricultural Plant Lifespan and Global Environmental Changes for Sustainable Green Technology.” Journal of Ecology & Natural Resources 5, no. 2: 1–18.

[pei370058-bib-0054] Qiu, W. , J. Kang , Z. Ye , et al. 2025. “Arbuscular Mycorrhizal Fungi Build a Bridge for Soybeans to Recruit *Pseudomonas putida* .” New Phytologist 246: 1276–1292. 10.1111/nph.70064. 40105301

[pei370058-bib-0055] Ramírez Caro, M. I. , I. Bennett , and N. Malajczuk . 2011. “Establishment of Endomycorrhizal Fungi on Micropropagated Teak (*Tectona grandis* L.f.).” BMC Proceedings 5, no. Suppl 7: P149. 10.1186/1753-6561-5-S7-P149.

[pei370058-bib-0056] Salvioli, A. , I. Zouari , M. Chalot , and P. Bonfante . 2012. “The Arbuscular Mycorrhizal Status has an Impact on the Transcriptome Profile and Amino Acid Composition of Tomato Fruit.” BMC Plant Biology 12: 44. 22452950 10.1186/1471-2229-12-44 PMC3362744

[pei370058-bib-0057] Santander, C. , R. Aroca , J. M. Ruiz‐Lozano , et al. 2017. “Arbuscular Mycorrhiza Effects on Plant Performance Under Osmotic Stress.” Mycorrhiza 27: 639–657. 10.1007/s00572-017-0784-x. 28647757

[pei370058-bib-0058] Sardans, J. , H. Lambers , C. Preece , A. F. Alrefaei , and J. Penuelas . 2023. “Role of Mycorrhizas and Root Exudates in Plant Uptake of Soil Nutrients (Calcium, Iron, Magnesium, and Potassium): Has the Puzzle Been Completely Solved?” Plant Journal 114, no. 6: 1227–1242. 10.1111/tpj.16184 36917083

[pei370058-bib-0059] Schubert, R. , S. Werner , H. Cirka , et al. 2020. “Effects of Arbuscular Mycorrhization on Fruit Quality in Industrialized Tomato Production.” International Journal of Molecular Sciences 21, no. 19: 7029. 10.3390/ijms21197029. 32987747 PMC7582891

[pei370058-bib-0060] Shalaby, O. A. , and M. E. S. Ramadan . 2024. “Mycorrhizal Colonization and Calcium Spraying Modulate Physiological and Antioxidant Responses to Improve Pepper Growth and Yield Under Salinity Stress.” Rhizosphere 29: 100852.

[pei370058-bib-0061] Shen, C. , B. F. Huang , Q. Liao , K. F. Chen , J. L. Xin , and Y. Y. Huang . 2025. “Uncovering Differences in Cadmium Accumulation Capacity of Different *Ipomoea aquatica* Cultivars at the Level of Root Cell Types.” Horticulture Research 12: uhaf077. 40297020 10.1093/hr/uhaf077 PMC12036322

[pei370058-bib-0062] Smith, S. E. , and F. A. Smith . 2011. “Roles of Arbuscular Mycorrhizas in Plant Nutrition and Growth: New Paradigms From Cellular to Ecosystem Scales.” Annual Review of Plant Biology 62: 227–250. 10.1146/annurev-arplant-042110-103846. 21391813

[pei370058-bib-0063] Tang, H. , M. U. Hassan , L. Feng , et al. 2022. “The Critical Role of Arbuscular Mycorrhizal Fungi to Improve Drought Tolerance and Nitrogen Use Efficiency in Crops.” Frontiers in Plant Science 6: 919166. 10.3389/fpls.2022.919166. PMC9298553 35873982

[pei370058-bib-0064] Tian, C. , B. Kasiborski , R. Koul , P. J. Lammers , H. Bücking , and Y. Shachar‐Hill . 2010. “Regulation of the Nitrogen Transfer Pathway in the Arbuscular Mycorrhizal Symbiosis: Gene Characterization and the Coordination of Expression With Nitrogen Flux.” Plant Physiology 153: 1175–1187. 10.1104/pp.110.156430. 20448102 PMC2899933

[pei370058-bib-0065] Ullah, F. , H. Ullah , M. Ishfaq , S. L. Gul , T. Kumar , and Z. Li . 2023. “Improvement of Nutritional Quality of Tomato Fruit With *Funneliformis mosseae* Inoculation Under Greenhouse Conditions.” Horticulturae 9, no. 4: 448. 10.3390/horticulturae9040448.

[pei370058-bib-0066] Ullah, F. , H. Ullah , M. Ishfaq , et al. 2023. “Genotypic Variation of Tomato to AMF Inoculation in Improving Growth, Nutrient Uptake, Yield, and Photosynthetic Activity'.” Symbiosis 92, no. 1: 111. 10.1007/s13199-023-00961-5.

[pei370058-bib-0067] Wakeel, A. , and M. Ishfaq . 2016. “Promoting Precise and Balanced Use of Fertilizers in Pakistan at Farm‐Gate Level.” Electronic International Fertilizer Correspondent (e‐Ifc) 1, no. 47: 20–25.

[pei370058-bib-0068] Wakeel, A. , and M. Ishfaq . 2022. Potash Use and Dynamics in Agriculture. 1st ed. Springer. 10.1007/978–981–16-6883-8.

[pei370058-bib-0069] Wang, L. , B. Song , M. Ishfaq , and X. Zhao . 2025. “Optimization of Nitrogen Fertilizer Application Enhanced Sugar Beet Productivity and Socio‐Ecological Benefits in China: A Meta‐Analysis.” Soil and Tillage Research 251: 106547. 10.1016/j.still.2025.106547.

[pei370058-bib-0070] Yadav, M. , P. Gupta , and C. S. Seth . 2022. “Foliar Application of α‐Lipoic Acid Attenuates Cadmium Toxicity on Photosynthetic Pigments and Nitrogen Metabolism in *Solanum lycopersicum* L.” Acta Physiologiae Plantarum 44: 112. 10.1007/s11738-022-03445-z.

[pei370058-bib-0071] Yang, S. , W. Wu , S. Liu , et al. 2025. “Seaweed Extract Combined With Boron Promotes the Growth of Sugar Beet by Improving the Photosynthetic Performance Under Boron Deficiency.” Physiologia Plantarum 177, no. 2: e70195. 10.1111/ppl.70195. 40175648

[pei370058-bib-0072] Yokamo, S. , M. Irfan , W. Huan , et al. 2023. “Global Evaluation of Key Factors Influencing Nitrogen Fertilization Efficiency in Wheat: A Recent Meta‐Analysis (2000‐2022).” Frontiers in Plant Science 14: 1272098. 10.3389/fpls.2023.1272098. 37965011 PMC10642427

[pei370058-bib-0073] Yoshida, S. , D. A. Forno , J. H. Cock , and K. A. Gomez . 1976. Laboratory Manual for Physiological Studies of Rice, 3rd ed, 14e16. International Rice Research Institute.

[pei370058-bib-0074] Zaheer, M. S. , N. Aijaz , A. Hameed , et al. 2024. “Cultivating Resilience in Wheat: Mitigating Arsenic Toxicity With Seaweed Extract and *Azospirillum brasilense* .” Frontiers in Microbiology 15: 1441719. 39228378 10.3389/fmicb.2024.1441719 PMC11368767

[pei370058-bib-0075] Zaheer, M. S. , H. H. Ali , S. Manoharadas , et al. 2024. “Exploring the Impact of Titanium Dioxide Nanoparticles (nTiO_2_) at Varied Concentrations in Combination With *Azospirillum brasilense* on Wheat Growth and Physiology.” Journal of King Saud University, Science 36: 103189.

[pei370058-bib-0076] Zhang, W. , Y. Chen , Z. Guan , et al. 2025. “Structural Insights Into the Mechanism of Phosphate Recognition and Transport by XPR1.” Nature Communications 16, no. 1: 18. 10.1038/s41467-024-55471-9 PMC11696373 39747008

